# Antepartum depressive and anxious symptoms: Association with physiological parameters of the newborn

**DOI:** 10.1192/j.eurpsy.2021.478

**Published:** 2021-08-13

**Authors:** D. Pereira, A.T. Pereira, J. Azevedo, S. Xavier, M.J. Soares, N. Madeira, A. Macedo

**Affiliations:** 1 Institute Of Psychological Medicine, Faculty Of Medicine, University of Coimbra, coimbra, Portugal; 2 Psychiatry Department, Centro Hospitalar e Universitário de Coimbra, Coimbra, Portugal; 3 Institute Of Psychological Medicine, Faculty Of Medicine, University of Coimbra, Coimbra, Portugal

**Keywords:** Anxiety, Depression, perinatal, Psychological disorders

## Abstract

**Introduction:**

The Perinatal period is a time of vulnerability for developing psychiatric disorders of higher prevalence in the female gender - depression and anxiety^1^. Numerous authors have proposed that maternal psychological factors could influence pregnancy course and the well-being of mother and newborn^2^.

**Objectives:**

To explore the relationship between perinatal psychological disorder and physiological parameters evaluated at birth, such as the Apgar Index (AI; 1, 5 and 10 minutes), head circumference, weight, length and age.

**Methods:**

533 women answered, in the second trimester of pregnancy (16.98±4.83 weeks of gestation), several questions about psychosocial variables, the Perinatal Depression Screening Scale^3^ and the Perinatal Anxiety Screening Scale^4^. Of these, 208 (39.0%) women were interviewed with the Diagnostic Interview for Psychological Distress^5^. Newborn physiological parameters were obtained from electronic health records.

**Results:**

AI was significantly (p<.01) and moderately (r≈.25) correlated with maternal anxious symptomatology, and with the experience of a stressful event in the last year (only AI 1 minute). Newborns of women with clinically relevant anxious symptomatology (>cutoff point, 14.6%) had significantly lower AI (p<.05), which was also observed in newborns of women who considered having had a stressful event (only AI 1 minute). Women’s newborns with maternal anxiety disorders during pregnancy (5.3%), had significantly lower values in AI, head circumference, weight and age of birth. Regression analyses showed that anxiety in pregnancy (symptoms and/or diagnoses) is a predictor of newborn physiological parameters, explaining significant percentages(r≈22%; p<.05) of its variability.

**Conclusions:**

Early detection of psychological disorders in pregnancy, namely anxiety, is determinant to prevent adverse neonatal outcomes.

**Disclosure:**

No significant relationships.

